# District nurses’ experiences of caring for leg ulcers in accordance with clinical guidelines: a grounded theory study

**DOI:** 10.1080/17482631.2017.1355213

**Published:** 2017-07-27

**Authors:** Annica Lagerin, Ingrid Hylander, Lena Törnkvist

**Affiliations:** ^a^ Department of Neurobiology, Care Sciences and Society, Division of Family medicine, Karolinska Institute, Stockholm, Sweden; ^b^ Academic Primary HealthCare Centre, Karolinska Institute, Stockholm, Sweden

**Keywords:** Clinical nursing research, grounded theory method, guideline adherence, primary health care, district nurse

## Abstract

This qualitative study used the grounded theory method to investigate district nurses’ experiences of caring for leg ulcers in accordance with clinical guidelines at seven primary health care centres in Stockholm, Sweden. Group interviews were conducted with 30 nurses. The results describe how district nurses strive to stay on track in order to follow clinical guidelines and remain motivated despite prolonged wound treatment and feelings of hopelessness. Three main obstacles to following the guidelines were found. District nurses used compensating strategies so the obstacles would not lead to negative consequences. If the compensating strategies were insufficient, perceived prolonged wound treatment and feelings of hopelessness could result. District nurses then used motivating strategies to overcome these feelings of hopelessness. Sometimes, despite the motivating strategies, treatment in accordance with guidelines could not be achieved. With some patients, district nurses had to compromise and follow the guidelines as far as possible.

## Introduction

A number of clinical practice guidelines aim to ensure optimal leg ulcer treatment (SIGN, 2010; WOCN, 2013). Research shows, though, that health care professionals do not always treat ulcers in line with clinical guidelines (Edwards et al., 2013; Lagerin, Nilsson, &Törnkvist, 2007), and previous research shows that a number of obstacles contribute to the problem. However, little is known about how nurses attempt to overcome these obstacles. Such information is important to increase our understanding of nurses’ clinical practice and identify ways to help improve leg ulcer management in primary health care.

The issue is increasingly relevant since older age is the most important risk factor for developing an active leg ulcer and the proportion of older people in the population is growing (Christensen, Doblhammer, Rau, & Vaupel, 2009). Older people often have several chronic diseases that can cause leg swelling, such as diabetes, heart disease and vascular diseases (Forssgren, Fransson, & Nelzén, 2008). Thus, the number of patients who have complex needs (Hellström, Nilsson, Nilsson, & Fagerström, 2016; Upton, Andrews, & Upton, 2014) that can negatively influence the development of leg ulcers is increasing (Hjort & Gottrup, 2010).

Leg ulcers are wounds below the knee that have not healed in 6 weeks (Nelson, 2011; The Handbook for Healthcare, 2016). Venous leg ulcers are the most commonly observed type of hard-to-heal ulcer (Nelson, 2011). Patients with leg ulcers are typically treated in primary health care (Hellström et al., 2016; Weller & Evans, 2012). General practitioners (GPs) normally set diagnoses (Sinha & Sreedharan, 2014) and are involved in treatment decisions, and nurses are usually responsible for dressing application, compression bandaging, and patient education (Kapp & Miller, 2015; Weller & Evans, 2012).

Sweden has online guidelines about the care of lower leg wounds (The Handbook for Healthcare, 2016). Such guidelines have been available to medical professionals since at least 2002.The main principles of wound care presented in the guidelines, which are based on the European Wound Management Association’s position documents, have not changed since that time (EWMA, 2002-2008). However, the web platform has been improved to provide more information (for example, it now includes short films illustrating treatment methods). Wound care techniques and materials have also undergone development. Swedish guidelines recommend that the cause of the ulcer should be diagnosed by a doctor and this diagnosis used to guide further treatment. The nurse is responsible for patient care, prevention of ulcers and ulcer complications, wound treatment, selection of appropriate wound dressings, compression treatment, informing the patient about the treatment and keeping the electronic patient record (EPR) up to date (The Handbook for Healthcare, 2016).

In Sweden, district nurses (DNs) are specialist nurses who work in primary care. They share the responsibility for patient care with other professionals, such as GPs, registered nurses, assistant nurses and medical social workers (Lagerin et al., 2007; Strandberg, Ovhed, Borgquist, & Wilhelmsson, 2007). DNs often treat patients with leg ulcers, both at health care centres and in home care (Friman, Klang, & Ebbeskog, 2010). Since healing is often slow and recurrence rates are high, treatment requires a great deal of nursing time and a large amount of dressing materials (Öien & Forssell, 2013). Nurses’ time accounts for the major portion of the total cost of treating leg ulcers (Öien, Forssell, & Ragnarson Tennvall, 2016).

Extensive research on difficulties in wound care shows that some patients are treated incorrectly or are treated without having been diagnosed (Weller & Evans, 2012). Reasons may include insufficiencies in the collaboration between DNs and GPs (Friman et al., 2010; Öien & Forssell, 2013) such as poor communication and poor coordination (Silva, Jesus, Merighi, & Oliveira, 2014); lack of support for best practice from the management of primary health care centres (Friman et al., 2010); and incomplete/inconsistent documentation (Khalil, Cullen, Chambers, Steers, & Walker, 2014).

Similarly to previous researchers (Edwards et al., 2013; Weller & Evans, 2012), in an earlier study, the current research group found that despite specific training in guideline-based treatment of leg ulcers, DNs did not entirely follow the recommended guidelines (Lagerin et al., 2007). Although obstacles to following guidelines for leg ulcer treatment have been scrutinized in earlier studies, little is known about how nurses struggle with these obstacles. Thus a natural next step was to investigate DNs’ main concerns about providing treatment in accordance with guidelines and what they do to cope with these concerns. This study thus aimed to investigate DNs’ experiences of caring for leg ulcers in accordance with clinical guidelines.

## Material and methods

### Study design

The grounded theory method (GTM) was chosen for data collection and analysis (Glaser & Strauss, 1967; Hylander, 2003). GTM is particularly useful when little is known about the field of interest or when a new perspective on a field is called for. Additionally, research using GTM typically focuses on social processes in participants’ natural settings and aims to form concepts and construct theories, which was also the aim of this study.

### Setting and participants

The study was conducted between 2006 and 2015 at seven primary health care centres that had purchaser-provider contracts with the Stockholm County Council to provide primary health care to residents. There were three inclusion criteria: being a DN, working at one of the seven participating centres and having clinical experience treating patients with leg ulcers. DNs and their managers received oral and written information about the study and an invitation to participate. A total of 30 DNs gave written consent and participated; all were women.

### Procedure

The corresponding author (AL) used a semi-structured interview guide to conduct group interviews at the DNs’ workplaces to obtain in-depth and varied data about DNs’ experiences of caring for patients with leg ulcers. AL discussed her impressions after each group interview with an observer (a DN familiar with group interview methods). For each interview, these impressions were noted in memos. The interviews were audio recorded and ranged in length from 40 to 50 min. The recordings were transcribed verbatim.

### The interview guide

An interview guide with open-ended questions was developed (Patton, 2002). Open-ended questions initially used during the interviews included “What is the daily health care situation like for DNs caring for patients with leg ulcers?”, “How do you work?”, and “How do you get patients to participate in treatment?” As analysis progressed and new issues emerged, questions in the interview guide became more focused. Examples of more focused questions included “What do you do when wounds don’t heal?” and “What do you do if patients won’t follow your advice?”

### Theoretical sampling

Seven group interviews were conducted (each DN participated in one group interview). In accordance with GTM, we used theoretical sampling (Charmaz, 2006; Hylander, 2003). That is, when questions emerged from the analysis of previous data, the authors changed the interview guide to focus on these new questions and then interviewed new groups. Five group interviews with 18 DNs were conducted between 2006 and 2007. The first round of data collection included three group interviews with two, four and four participants from centres of Stockholm County Council area. The second round of data collection included two new groups with three and five participants from centres in a different region of the Stockholm County Council area. These DNs were interviewed with a slightly different interview guide that focused on obstacles to caring and strategies for overcoming these obstacles. To increase the variation of data and saturate the categories, AL conducted two additional group interviews with 12 DNs (four and eight participants) in January 2015.

### Analysis

To develop a substantive theoretical model, data collection and analysis were conducted in parallel. The analysis followed the steps outlined by Hylander (2003). Constant comparison was conducted in stages, between indicators and codes, between codes, between codes and categories, between categories, and finally between the core process and larger excerpts from the interviews. In a first stage of analysis, *open coding*, the transcriptions of the three first group interviews were read line by line and the data were analysed and coded. Similarities and differences between codes were identified, and similar codes were sorted into the same category, a process that resulted in 13 preliminary categories (e.g., “no good routines”, “patients want to make up their own mind”, “compromise”, and “a wide variety of bandages”).

In a second stage, *focused coding* was used to fill the categories and develop aspects and dimensions of categories. The preliminary categories were reduced to eight conceptualized main categories. In the third stage of analysis, the relationships between categories were identified via theoretical codes such as “obstacles” (e.g., unsupportive wound treatment organization) and “strategies for overcoming obstacles”. Finally, in the fourth stage of analysis, the core process was elaborated. This core process tied all the categories together and described how DNs *strive to stay on track*, i.e., to follow guidelines and stay motivated in order to reach the goal of healed ulcers despite prolonged wound treatment and feelings of hopelessness. The researchers collected data until they estimated that saturation had been reached, i.e., until categories were filled and no new categories were found.

### Ethical considerations

The DNs and their managers at the primary health care centres received verbal and written information about the purpose of the study and a request that the health centre participate in the study. Informed consent was obtained from the individual participants after they had received the written and verbal information. Participants’ confidentiality was guaranteed, as was their anonymity in the presentation of findings. The study was approved by the Regional Ethical Review Board in Stockholm (Dnr 2010/566–31/5).

## Results

**Figure 1.**
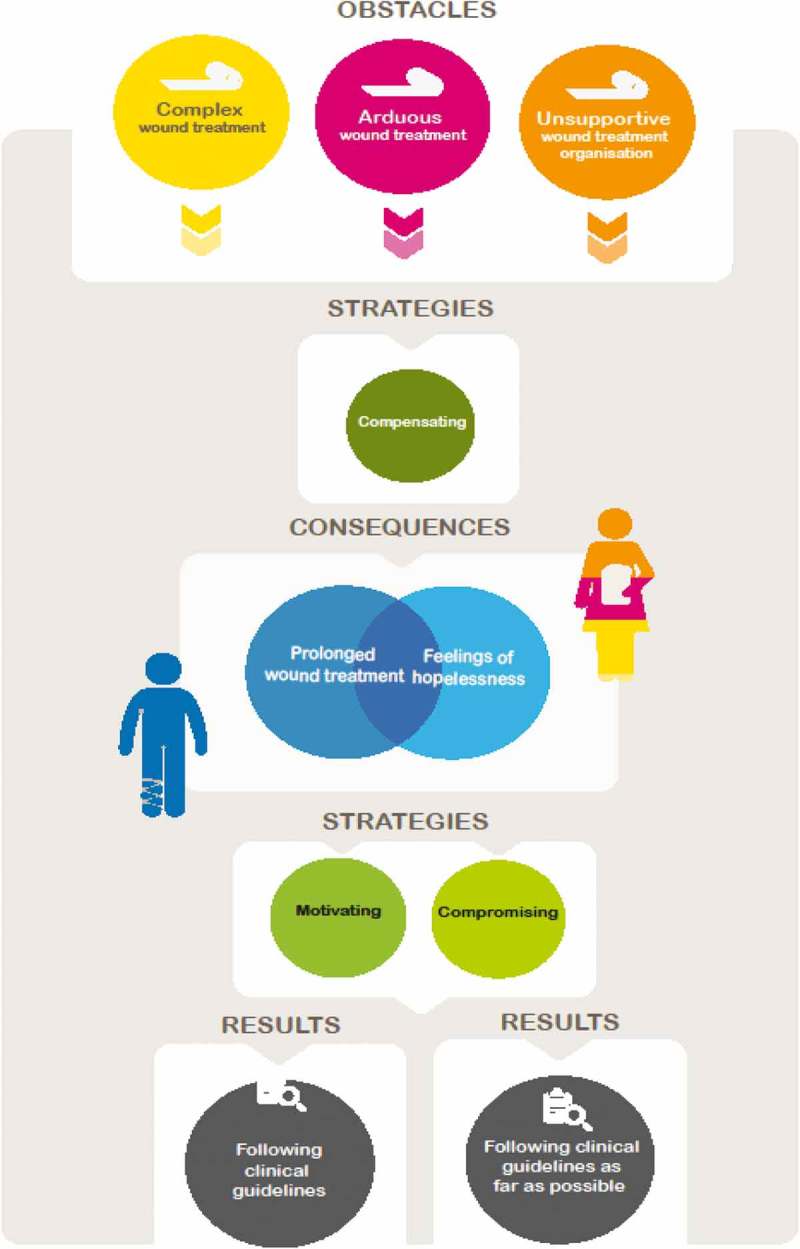
The core process: “District nurses strive to stay on track”.

The core process describes how DNs strive to stay on track; that is, to follow clinical guidelines and remain motivated despite prolonged wound treatment and feelings of hopelessness. DNs reported several obstacles to following the guidelines: complex wound treatment, arduous wound treatment, and an unsupportive wound treatment organization. These obstacles resulted in perceived prolonged treatment that led to feelings of hopelessness when DNs and/or patients saw no progress. However, DNs strived, via a set of specific strategies, to follow guidelines, stay motivated, and help patients remain motivated. They used compensating strategies to avoid consequences of the obstacles and motivating strategies to overcome feelings of hopelessness. Compensating and motivating strategies interacted to facilitate best practice. It was not possible for DNs to follow guidelines without strategies for remaining motivated, and it was not possible to remain motivated without striving to follow guidelines. Even if the DNs remained motivated and strived to follow the guidelines, they could not always achieve this goal. With some patients, DNs had to compromise and follow the guidelines as far as possible (i.e., provide leg ulcer care acceptable to the patient and the DN) (Figure 1).

The following section begins with a description of each obstacle to adhering to clinical guidelines and describes the sub-categories, i.e., the aspects of each obstacle. The descriptions are followed by information on DNs’ strategies to avoid consequences of these obstacles. The section then covers prolonged wound treatment and the feelings of hopelessness that occur when DNs and patients do not perceive progress in treatment. DNs’ strategies for overcoming feelings of hopelessness are then described. Finally, the strategy DNs used to compromise when they are not able to completely follow guidelines is presented (Table I).

**Table I.** Obstacles experienced and strategies used by DNs as they strive to stay on track in order to follow clinical guidelines for leg ulcer care.

**Obstacles to following the guidelines**		
**Obstacle 1: Complex wound treatment** **Challenging collaboration with GPs** **Complex patient pathology** Old age Undernutrion Multiple diseases Disability Mental illness Cognitive problems **Complex wound assessment**	**Obstacle 2: Arduous wound treatment** **Difficult working environment** Lack of necessary equipment Poor lighting Unhygienic situations in some patients’ homes Physically demanding working positions **Troublesome treatment for patients** Physically troublesome Psychologically troublesome Financially troublesome	**Obstacle 3: Unsupportive wound treatment organization** **Unevenly distributed competence in and experience of leg ulcer treatment** **Unclear responsibility for individual patients’ wound treatment** Several DNs might treat the same leg ulcer Unclear responsibility for individual treatment plans **No easy access to treatment plans** No access to EPRs in patients’ homes Difficulty finding important information about treatment plans in EPRs **Unclear policy for ordering materials**
**Compensating strategies for avoiding consequences of the obstacles**		
**Compensating strategies DNs use in order to follow guidelines when treatment is complex** **Communicating proactively with GPs** Trying to catch the GP in the corridor Taking the initiative to make GP appointments for their clinical patients Taking home-care patients to the health centre Informing the GPs when the time has come to take action Using technology in innovative ways **Updating their knowledge and skills** Learning on their own Seeking information from companies that make wound care products Talking with professionals who work at clinics that specialize in wound care	**Compensating strategies DNs use to follow guidelines even when treatment is arduous** Collaborating with others, such as assistant nurses and home help service personnel Informing patients about treatment	**Compensating strategies DNs use in order to follow guidelines when the wound treatment organization is unsupportive** **Planning in order to achieve care continuity** Planning so that one DN has the main responsibility for each patient Making appointments with patients to avoid drop-in visits Creating individual treatment plans in the EPR For home care patients, creating an additional individual wound treatment plan (with key words) that is kept in the patient’s home **Clarifying policy for ordering dressing material** **Mentoring less experienced colleagues**
**Perceived prolonged wound treatment as consequences of the obstacles** Patients’ feelings of hopelessness DNs feelings of hopelessness		
**Consequences of complex wound treatment** Lack of up-to-date information Lack of aetiological diagnoses Patients’ lack of ability to cooperate Time-consuming treatment	**Consequences of arduous wound treatment** Experienced DNs might stop working with leg ulcers in home care Patients might not adhere to clinical guidelines	**Consequences of an unsupportive wound treatment organization** Some patients do not receive leg ulcer care in accordance with guidelines Patients may be treated by different nurses on different occasions DN does the same as the former DN DN makes own treatment choice Difficult to choose among different dressing materials Treatment plans were not accessible to DNs
**Strategies for overcoming feelings of hopelessness**		
**Motivating strategies** **Motivating oneself** **Motivating patients** Establishing a trusting relationship with the patient Evoking hope Involving the patient and/or family members in the care process Taking photos of the wound at regular intervals **Compromising strategy**		

District nurse (DN), General Practitioner (GP), electronic patient record (EPR).

### Obstacle 1: complex wound treatment

The complexity of wound treatment constituted the first obstacle to adhering to guidelines. From the DNs’ perspective, aspects of this obstacle included: *challenging collaboration with GPs, complex patient pathology*, and *complex wound assessment.*

#### Challenging collaboration with GPs

To follow guidelines, DNs needed an aetiological diagnosis, which had to be made by a GP. Sometimes the DNs also needed to collaborate with GPs to assess and treat infections and pain. However, the DNs reported that many GPs did not have the time for the home visits that were sometimes necessary to diagnose or treat patients. The DNs said the GPs’ lack of time was due to the Swedish system for compensating health centres for patient care, which obligated GPs to prioritize short patient visits to the centres. Moreover, the DNs reported that some GPs’ competence in wound treatment was not sufficient to enable them to give the DNs the advice they needed. These challenges in collaborating with GPs resulted in few etiological diagnoses for patients in home care and constituted an obstacle to following the guidelines. One DN stated:

It’s hard to get them to a doctor … it’s a slow system. Or if a doctor should go home and look. We’re mostly the ones who take care of things in our own way, I think. That’s unfortunately the way it is in practice. (DN 10)

#### Complex patient pathology

It could also be difficult to follow guidelines because of complex patient pathology. Old age, undernutrition and multiple diseases, such as impaired circulation, diabetes, or other chronic diseases could negatively influence the wound-healing process and constitute a risk for prolonged treatment. “Many are very old; they have lousy circulation. They’re not fit for an operation. It’s just totally impossible to get the wound to heal”, one DN said (DN 19).

Disability, mental illness (including addiction), and cognitive problems could also enhance the complexity of treatment. DNs said that cognitive problems could lead to non-adherence to treatment. For instance, patients with cognitive problems might miss a planned health care visit or might not understand the importance of using a compression bandage. According to one DN, many patients with dementia took off their compression bandages because they were tight. DNs also said that they might be the only social contact that some lonely patients had, and in such cases, the patients might not want the ulcers to heal.

#### Complex wound assessment

Wound assessment is a complex procedure that includes multiple elements, such as assessing the aetiology of the wound; choosing a treatment, wound dressings and compression material; assessing preventive measures; creating individual treatment plans; and keeping information current in the EPR, including taking a photograph of the ulcer and writing a description of it. For DNs to be able to follow the guidelines, hindrances related to each aspect of the obstacle had to be overcome. Assessing the aetiology of the wound was difficult if collaboration with the GP was challenging. Choosing treatment, wound dressings, and compression material was complex because numerous innovative dressing and bandage materials were available. It was also difficult to keep up with new information from wound care companies. DNs described the whole process of wound assessment as a complex and time-consuming process and said that keeping up with it was difficult.

### Compensating strategies DNs use in order to follow guidelines when treatment is complex

*Communicating proactively with GPs* and *updating their knowledge and skills* were strategies DNs used to compensate for the challenges posed by complex treatment.

#### Communicating proactively with GPs

The nurses attempted to involve GPs actively in treatment. DNs needed diagnoses and treatment recommendations from GPs, and their strategies for meeting this need included trying to catch the GP in the corridor and taking the initiative to make GP appointments for their clinical patients (that is, those who came to the health centre for care). GPs often had difficulty scheduling home visits, so DNs sometimes helped by taking home care patients to the health centre. One DN said, “It’s easier for the doctor to visit the patient at the clinic than in the home” (DN 18). A further strategy was informing the GPs when the time had come to take action; for example, when it was time to refer the patient to a specialist for assessment. “We tell them what they should do. That’s easiest, because we’re usually better with wounds than what they are”, said one DN (DN 22). Some DNs also reported using technology in innovative ways to save time by avoiding the need for a visit to the health centre. For instance, a DN might photograph a leg ulcer with a mobile phone camera and show the photo to the GP to get a diagnosis.

#### Updating their knowledge and skills

Another strategy DNs used to follow guidelines despite complex treatment was learning on their own. That is, they sought information and understanding by reading new journal articles and other literature on wound care, participating in workshops and lectures and consulting each other. If they belonged to a health care team at the centre where they worked, they might also discuss wound care with this team.

We don’t have anything special, but every year a few [of us] get to go away to a course, and we tell the others about it. And we discuss ulcers. If you have an ulcer that doesn’t heal, we talk about it with a colleague. I’ve tried that … there are discussions all the time. (DN 20)

Seeking information from companies that make wound care products and talking with professionals who work at clinics that specialize in wound care were additional strategies DNs used to follow guidelines even when treatment was complex.

### Obstacle 2: arduous wound treatment

Aspects of arduous wound treatment that DNs perceived as barriers to following guidelines included a *difficult working environment* in home care and *troublesome treatment for patients*.

#### Difficult working environment

DNs said that there was sometimes a lack of necessary equipment, such Doppler ultrasound or the wound dressings and compression materials that they preferred or that were called for in the patient’s care plan. They also described poor lighting and unhygienic situations in some patients’ homes. Physically demanding working positions complicated DNs’ work with leg ulcer patients in home care. Such working positions could lead to back problems, particularly for older DNs. This, in turn, might make it difficult to continue working with leg ulcer patients in home care. One DN said, “The worst things in homes are the working positions and the light. You can’t see what the wound looks like. Some people keep it so dark at home. It’s really tricky. [I’m] thinking about getting a head lamp” (DN 10).

#### Troublesome treatment for patients

Treatment for leg ulcers could be physically, psychologically, and financially troublesome for patients. Physically, compression bandages can cause itching or aching, can feel too tight, and can stick to wounds. “It can hurt when we change a bandage, when we take off the dressing. Then the patient can be in a bad mood even if we try to be as gentle as possible”, said one DN (DN15). The treatment could also be psychologically troublesome. The DNs said that patients might become upset if bandages limited their mobility or made it difficult to get their feet into shoes. Wounds that exuded could also upset patients. Treatment could be financially troublesome if patients could not easily afford the expense of items such as new and larger shoes, medication, or extra home help service to assist them with putting on and taking off compression hosiery. One DN said, “… many have a hard time getting support stocking on. That’s a big problem if they don’t have any home help service” (DN 7).

### Compensating strategies DNs use to follow guidelines when treatment is arduous

To follow the guidelines for the treatment of leg ulcers, DNs compensated for the arduous aspects of treatment. Their strategies included *collaborating with others* and *informing patients about treatment*.

#### Collaborating with others

DNs collaborated with other DNs or assistant nurses at their workplace to manage tasks such as heavy lifting. They also collaborated with wound care clinics at hospitals and with home help service personnel. For example, home help service personnel could aid the DNs by taking off a patient’s bandages and showering the affected leg. “You involve home help services. The patient can go outside and move. Eat better—like all that. The obvious things round about” (DN 20). Another DN said:

When you meet someone from home help service, you say that they can take the bandage off when they shower. Many patients, especially when they have large leg ulcers, appreciate it if they can shower—afterward they feel a little better (DN 15).

#### Informing patients about treatment

DNs saw several reasons why patients did not adhere to guideline-based treatment. For example, they reported that some patients resisted switching from shoes they were used to wearing to shoes they needed in order to use compression bandages. To get patients to adhere to treatment, DNs informed them about why their legs were swollen and how compression hosiery helps the healing process. They also gave patients advice about what they could do to promote healing. “I talk about movements, pedalling the sewing machine, you always do” (DN 1).

### Obstacle 3: unsupportive wound treatment organization

According to DNs, unsupportive wound treatment organization constituted an important obstacle to following guidelines. DNs said that health centre managers did not prioritize leg ulcer treatment and that it was difficult to get managers to listen when they suggested organizational improvements. They described four main aspects of an unsupportive wound treatment organization: *unevenly distributed competence in and experience of leg ulcer treatment, unclear responsibility for individual patient’s wound treatment, unclear policy for ordering materials*, and *no easy access to treatment plans*.

#### Unevenly distributed competence in and experience of leg ulcer treatment

When experienced DNs at health centres were replaced by novice nurses or nurses with no specialist education in district nursing, competence and routines were lost. DNs said centres sometimes relied heavily on inexperienced DNs or nurses who lacked competence in leg ulcer treatment. One DN said, “Anyone has come—for example, newly trained nurses” (DN 30). Some of the DNs also reported that their leg ulcer and wound treatment education was inadequate, as classes on these topics were not mandatory in their specialist educational.

#### Unclear responsibility for individual patients’ wound treatment

It was sometimes unclear which DN was responsible for the wound treatment of individual patients visiting primary health care centres. When DNs at the centre rotated on the job, sometimes several treated the same leg ulcer. Even if DNs wanted to follow up a specific patient’s leg ulcer, it was not always possible. One DN said, “Then I’m on the phone on telephone duty, and then I can’t run off and look. It’s a huge deficiency” (DN 27). Rotation was not such an important barrier in home health care, as DNs had their own home care districts and were responsible for the care of patients in that district. Responsibility for individual treatment plans was also unclear. In other words, in some cases, no one took the responsibility for writing a treatment plan.

#### No easy access to treatment plans

When DNs worked with home care patients, they could not consult the EPR and thus had no easy access to treatment plans. Some DNs also had difficulty with EPRs at the workplace. At the time of the study, primary health care centres in Stockholm County had just adopted a new EPR system, and some DNs found it difficult to work with, especially under time pressure. They also found it difficult to find important information in treatment plans in EPRs. One DN said, “There might be a hundred notes like that. Then there’s a note in the middle where there’s really something of value, and it’s very hard to find it” (DN 9).

#### Unclear policy for ordering materials

DNs experienced a lack of routines and policies for ordering dressing and compression materials. There are many alternatives on the market, which means that choosing what to order can be difficult for DNs. Unclear ordering routines and policies could lead to a confusing variety of dressing materials at the clinic. One DN said, “There are loads of bandages. It’s difficult for DNs to agree. Everyone wants their own. Then there’s a huge supply to take from” (DN 1).

### Compensating strategies DNs use in order to follow guidelines when the wound treatment organization is unsupportive

*Planning in order to achieve continuity of care, clarifying policy for ordering dressing materials*, and *mentoring less-experienced colleagues* were strategies used by the DNs to compensate for an unsupportive wound treatment organization.

#### Planning in order to achieve care continuity

One strategy for achieving care continuity for patients who came to the health centre was planning so that one DN had the main responsibility for each patient. “We try [to make sure] that it’s the same district nurse that cares for the patient [and] that follows the same ulcer as far as possible” (DN 11). Other strategies included making appointments with patients to avoid drop-in visits and creating individual treatment plans in the EPR for each patient. These plans included a nursing diagnosis, treatment goal, planned nursing care and key words related to the guidelines. The key words helped structure the nursing care documentation and made it easier for the nurses to search the EPRs for the information they needed. Because DNs did not have access to the EPR when they were working with home care patients, creating an additional wound treatment plan (with key words) kept in the patient’s home was another strategy they used to achieve care continuity.

#### Clarifying policy for ordering dressing material

DNs suggested that the centres should work on clarifying their policy for ordering dressing materials. The DNs thought that a few well-selected alternative dressings with which they were familiar would make it simpler to select material.

#### Mentoring less-experienced colleagues

DNs suggested that newly employed nurses might benefit from mentoring, either by DNs or others with deeper knowledge of leg ulcer treatment.

### Perceived prolonged wound treatment as a consequence of obstacles

Without compensating strategies for overcoming obstacles posed by complex, arduous treatment and by an unsupportive wound treatment organization, DNs and patients perceived wound treatment as prolonged. When they saw no wound healing progress, such prolonged treatment led to feelings of hopelessness in both patients and DNs.

#### Consequences of complex wound treatment

When DNs could not compensate for the complex nature of treatment, they lacked up-to-date information, and their patients typically lacked aetiological diagnoses. Moreover, treatment complexity could contribute to patients’ lack of ability to cooperate and to time-consuming treatment.

#### Consequences of arduous wound treatment

When the DNs could not compensate for the arduous nature of treatment, experienced DNs might stop working with leg ulcers in home care and patients might not adhere to clinical guidelines.

#### Consequences of an unsupportive wound treatment organization

When DNs could not compensate for an unsupportive wound treatment organization, some patients might not receive leg ulcer care in accordance with guidelines. Furthermore, patients treated by different nurses on different occasions would lack continuity of care. Sometimes the DN who treated the patient might simply do the same as the former DN without checking to make sure this treatment accorded with guidelines. In other instances, a DN might not follow the same procedure as the previous DN but rather make her own treatment choice and thereby deviate from the treatment plan. Unclear responsibility for writing the treatment plan could mean that treatment plans were not accessible or did not exist.

### Feelings of hopelessness

When there was no progress and treatment was prolonged, DNs and patients experienced feelings of hopelessness.

#### Patients’ feelings of hopelessness

When they experienced some or all of the consequences of the obstacles and saw no progress in wound healing, some patients could lose their motivation to adhere to treatment. The DNs reported that some patients said they were too old for further investigation or treatment of their leg ulcers in specialist care (e.g., surgery). “They believe that the wound will never heal. They have given up”, said one DN (DN 12).

#### DNs’ feelings of hopelessness

Prolonged treatment and lack of progress could also cause DNs to lose motivation. The DNs described feelings of hopelessness when ulcers would not heal or when patients would not or could not adhere to treatment. One said:

Sometimes I can also think that it feels a little hopeless. You know that the patient has poor circulation because they’ve done a circulation evaluation, but you can’t offer the patient so much. They eat poorly and we try supplemental nutrition drinks but they don’t want to drink it and … it’s slow. It can be heavy. It feels like nothing is happening. (DN 25)

To overcome their own and/or the patients’ feelings of hopelessness, DNs used *motivating* and *compromising strategies*.

### Motivating strategies for overcoming feelings of hopelessness

Motivating strategies included *motivating oneself* and *motivating patients*.

#### Motivating oneself

Motivating oneself meant thinking positively, being patient, maintaining trust that the leg ulcer would heal, and seeing the treatment as an exciting challenge. One DN said that to be able to continue working as a DN, you had to enjoy treating ulcers. Others thought that it was not enough to enjoy treating leg ulcers. One emphasized the need for patience:

But many you treat for leg ulcers can be nice, and treating them can be nice both for you and for the patient if you have time. But for some patients, when you take the elevator up, you can feel that you do yoga breathing to calm down … to be a professional person before you ring the doorbell. It’s like that, but usually you can have a nice time. You can have very nice moments and get great relationships. (DN 19)

#### Motivating patients

Motivating patients consisted of *establishing a trusting relationship with the patient, evoking hope, involving the patient and/or family members in the care process*, and *taking photos of the wound at regular intervals.*

Establishing a trusting relationship was seen as key to motivation. One DN said:

It’s important to get to know the patient—that the patient trusts you and understands what you mean. The first time they might wonder what’s this crazy stuff [you’re doing], but then things go well and then they notice that it works, they heal. (DN 16)

Evoking hope was also important. One DN said, “But you encourage and say it can probably heal, you never know, you work for it” (DN 12). DNs involved patients and/or their family members in the care process in a variety of ways. For example, the DNs might ask a patient to bandage his or her own leg with a compression bandage or ask family members to help with tasks like choosing the right type of food, keeping track of meals and reminding patients to elevate swollen legs. Taking photos of the wound at regular intervals over time could show progress towards healing and thus motivate patients to adhere to treatment. A DN explained:

That’s something you should [do], photograph the ulcer a little more often or measure the ulcer. It also has … [to do] with encouragement. You gain some small centimetre there or a half centimetre. You gain something in any case. Something happens in any case. It’s hard to catch with the naked eye. I would like to use the camera a little more. (DN 11)

### Compromising to overcome feelings of hopelessness

Guidelines could only be followed to the extent the patient was motivated to follow them. If motivating strategies failed, DNs had to compromise with patients. One DN said, ‘If you’re too direct and determined, the patient can say, “then I don’t want anything at all” (DN 9). Sometimes DNs would adjust their choice of dressing material and compression bandages to coax the patients to use them. Compromising in this way meant that the DNs could not follow guidelines 100% but that they could adhere to a treatment plan that was acceptable to the patient and hopefully would lead to care that was good enough to achieve the goal of a healed ulcer (Table I).

## Discussion

This study resulted in a theoretical model that illustrates how DNs compensate for obstacles, motivate themselves and their patients, and compromise as they strive to follow clinical guidelines for leg ulcer care. Some of the aspects of the obstacles to following guidelines elucidated in this study have been described before. However, the study brings this information together in a model, and in addition, provides information on the strategies DNs use to avoid or overcome consequences of the obstacles.

If DNs did not use compensating strategies, the complex nature of treatment could lead to a lack of up-to-date information and a lack of aetiological diagnoses. The study identified challenging collaboration with GPs as an important aspect of complex treatment. Previous studies have found that barriers to following guidelines include lack of routines for cooperation between nurses and GPs (Eskilsson & Carlsson, 2010; Friman et al., 2010) and/or GPs’ lack of access to clinical guidelines and experience treating chronic leg ulcers (Cullen & Phillips, 2009; Graham, Harrison, Shafey, & Keast, 2003). DNs’ strategies for avoiding consequences of this obstacle included proactively informing GPs when it was time to take action, such as when a patient needed a referral to a dermatology clinic. However, Friman et al. (2011) also found that DNs thought they should be responsible for referring patients to specialist clinics, as they often knew more about leg ulcer management than GPs. This knowledge came from their experience of treating leg ulcers, which was more extensive than that of the GPs (Friman, Wahlberg, Mattiasson, & Ebbeskog, 2014). Giving DNs in Sweden this responsibility and possibly even the task of aetiological diagnoses of leg ulcers (after appropriate supplementary education) would be one way to cope with this challenge.

Others have reported a lack of support from the management of health care centres, a lack of management interest in developing nurses’ wound-management skills and a lack of interdisciplinary work in wound care (Friman et al., 2014; Silva et al., 2014). In the present study, DNs compensated for such aspects by working to achieve care continuity. Another aspect of unsupportive wound treatment organizations also noted by other researchers is a lack of nurses trained and experienced in leg ulcer treatment (Brown, 2014; Silva et al., 2014; Ylönen, Stolt, Leino-Kilpi, &Suhonen, 2014). Such a lack may lead to care that is not in accordance with guidelines. DNs in this study suggested that mentoring less experienced colleagues would be a way to compensate for unevenly distributed competence.

When DNs did not compensate for the combination of complex and arduous wound treatment and an unsupportive wound treatment organization, DNs could perceive treatment as prolonged, which in turn could lead to feelings of hopelessness. Others have also reported that nurses feel professional frustration if leg ulcers worsen or come back (Silva et al., 2014). In this study, DNs used self-motivating strategies to overcome their feelings of hopelessness. It is important to consider that the DNs themselves need to be motivated in order to compensate for the obstacles they face in providing guideline-based treatment. They must also feel motivated in order to motivate patients. Brown (2014) also found that staff have to be motivated to negotiate treatment and self-care strategies with patients who have leg ulcers (Brown, 2014). Eskilsson and Carlsson (2010) reported that nurses tried to find the energy to reach harmony in their work with leg ulcers through reflection, acceptance, and distance. The DNs in this study had similar self-motivating strategies, such as thinking positively and seeing the treatment as an exciting challenge. The issue of nurses’ motivation in the care of patients with leg ulcers is clearly important and in need of more attention in clinical practice and in research.

Some of the DNs said that when wound treatment was prolonged, patients could lose hope that their ulcers would heal. Ebbeskog and Ekman (2001) found a dialectical process of hope and despair in patients with leg ulcers whose healing process was lengthy (Ebbeskog & Ekman, 2001). On the one hand, the patients felt imprisoned; on the other, they hoped for healing and freedom from the burden of a sick body. Motivating strategies DNs in the current study used to help patients overcome feelings of hopelessness included creating trust. Trust is an important element in all nurse-patient relationships, particularly in work with older patients who have complex needs (Ebbeskog & Ekman, 2001; Upton et al., 2014). Evoking hope was another motivating strategy used by DNs. Other studies have also shown that when health care professionals instil hope, it can have a positive impact on patients’ ability to cope with leg ulcers (Salomé, de Almeida, & Ferreira, 2015).

The results of the current study indicate that compensating and motivating strategies sometimes had to be combined with compromising with patients. Compromising was an act of balance: DNs could not follow guidelines 100% but could adhere to a treatment plan that was acceptable to the patient and hopefully would lead to the goal of a healed ulcer. This finding raises the question of whether following guidelines always is the ultimate goal. Compensating and motivating can advance care to a certain point. However, taking patient-centred care seriously means listening to the patient’s perspective. If that perspective is that the patient doesn’t tolerate guideline-based care, DNs have to solve that dilemma, and in this study, they solved it by providing good-enough care.

### Methods used to show trustworthiness

One major strength of the present study was that participating DNs spoke openly about their experiences of treating patients with leg ulcers. Another was that the interactions between DNs in the group interview produced new data. The first author has professional experience as a DN, which facilitated data collection but could also lead to bias. To compensate, the first author collaborated closely with the other authors in the analysis of data. Quotations were used to elucidate the grounding of the data and strengthen trustworthiness. The GTM of constant comparison was used throughout the study. To determine the relevance of the findings, the theoretical model was presented and discussed with 10 DNs and one nurse who worked in home care (10 women and one man who had an average of 12 years of professional experience in district nursing. The respondents were not the same as the previously interviewed nurses. DNs answered four questions and confirmed that the main categories were comprehensible, that the model was a valid way of describing their main concern in striving to use clinical practice guidelines, and that the model was a useful tool in the care of patients with leg ulcers. Most of the DNs did not find the information new but thought that the model was a useful tool for reflecting about the problem of leg ulcer treatment in primary health care (Table II). The theoretical model can thus be regarded as consistent and clinically relevant.

**Table II.** Questions asked to validate the model “District nurses strive to stay on track—to follow clinical guidelines and remain motivated despite prolonged wound treatment and feelings of hopelessness”. Respondents: 10 district nurses and one home care nurse.

Questions	Yes (*n*)	No (*n*)	Don’t know (*n*)
1. Do you recognize the description of main categories in the model?	11	0	0
2. Is there anything new in the model?	2	9	0
3. Is the model clear and easy to understand?	11	0	0
4. Can you use the model?	9	1	1

The results are provided as the number (*n*) of answers.

### Limitations

Even if well-grounded in empirical data, a grounded theory is never more than a set of proposals amenable to application and testing in similar areas and contexts. The participants in this study came from an urban environment, which might have limited the variation in data and thus also the variation in obstacles and the strategies used to overcome them. Another limitation might be the long study period, since clinical practice may have differed over time. For instance, the DNs interviewed in the first round of data collection might have lacked knowledge about how to use computer software in the field. In some ways, however, the long study period was advantageous, as it gave us the opportunity to explore the same complex nursing care issues over time. The data gathered in the second round of data collection indicated that the DNs faced the same obstacles as they had in the first round of data collection. Furthermore, despite the long study period, the responding nurses found the theoretical model valid, relevant and useful.

### Conclusions

This study illustrates how DNs compensate to avoid consequences of obstacles, motivate themselves and their patients to overcome feelings of hopelessness, and compromise with patients as they strive to follow clinical practice guidelines. The theoretical model that resulted from the study illuminates the work DNs do to balance compensating, motivating and compromising in order to follow clinical guidelines as far as possible and provide care that is good enough.

The theoretical model elucidates the importance of DNs’ continued training in wound care but also their use of self-motivating strategies to overcome frustration when wound treatment is prolonged. All the obstacles described in this study need attention; for instance, primary health care centre managers can support nurses via new routines, reflection and continuing education in wound management. However, the main contribution of this study is the description of the strategies DNs use to avoid and overcome the consequences of obstacles. This model can be used as a basis for discussions and changes in nursing practice and as a tool in university and continuing educational programmes about leg ulcer treatment. It can also help inform the organization of leg ulcer treatment at health care centres. Additionally, future studies could test whether the model is also applicable to nursing care for other chronic conditions in the community.

The study has potential implications for health care policy. Firstly, the findings indicate that it would be wise to include leg ulcer treatment as a routine component of specialist education for DNs. Secondly, it seems important for county councils to ensure that DNs and GPs have the opportunity for continuing education in leg ulcer treatment. Continuing interprofessional education is particularly relevant, given the need to improve collaboration between DNs and GPs in the treatment of patients with leg ulcers. Thirdly, the findings suggest that policy makers should engage in a discussion about the distribution of responsibilities between professions; care for patients might be enhanced if DNs were given the right to refer patients to specialist care for leg ulcers. Furthermore, the model can be tested in further quantitative studies to investigate whether use of the strategies is associated with better patient outcomes.
